# The emerging clinical role of wearables: factors for successful implementation in healthcare

**DOI:** 10.1038/s41746-021-00418-3

**Published:** 2021-03-10

**Authors:** Matthew Smuck, Charles A. Odonkor, Jonathan K. Wilt, Nicolas Schmidt, Michael A. Swiernik

**Affiliations:** 1 Division of Physical Medicine & Rehabilitation, Department of Orthopaedic Surgery, Stanford University, Stanford, CA USA; 2 Department of Orthopaedics and Rehabilitation, Yale University School of Medicine, New Haven, CT USA; 3 Chief Technology Officer of Innovation, Ochsner, Ochsner Health System, New Orleans, LA USA; 4 Head of Healthcare Products, Digital Health, Nokia Technologies, Cambridge, MA USA; 5 Medical Informatics, Kaiser Permanente Southern California, Pasadena, CA USA

**Keywords:** Health care, Diseases

## Abstract

Wearable technologies promise to redefine assessment of health behaviors, yet their clinical implementation remains a challenge. To address this gap, two of the NIH’s Big Data to Knowledge Centers of Excellence organized a workshop on potential clinical applications of wearables. A workgroup comprised of 14 stakeholders from diverse backgrounds (hospital administration, clinical medicine, academia, insurance, and the commercial device industry) discussed two successful digital health interventions that involve wearables to identify common features responsible for their success. Seven features were identified including: a clearly defined problem, integration into a system of healthcare delivery, technology support, personalized experience, focus on end-user experience, alignment with reimbursement models, and inclusion of clinician champions. Health providers and systems keen to establish new models of care inclusive of wearables may consider these features during program design. A better understanding of these features is necessary to guide future clinical applications of wearable technology.

## Introduction

Wearable technology, also known as “wearable devices” or simply “wearables”, generally refers to any miniaturized electronic device that can be easily donned on and off the body, or incorporated into clothing or other body-worn accessories^1^. While wearables have established utility in the fitness, gaming and entertainment industries, their role in the healthcare environment remains less clear^2^. To date most commercially available wearables are limited in scope, tracking one or two health-related variables, and have yet to produce accurate measurement of many markers of health status that they attempt to assess such as heart rate variability, nutrition, and mood^3–5^. To the extent that wearables overcome these limitations, they hold much promise towards expanding the clinical repertoire of patient-specific measures, and they are considered an important tool for the future of precision health.

Physical activity information is perhaps the most common measurement provided by current wearable devices, thus it serves as a useful example to review the opportunities and difficulties facing digital health development using wearables. Physical activity is a well-established marker of current health status and future health risks, it is a useful estimate of real-life functional performance^6^, and it has been tracked in health research using body-worn sensors for many decades^7–10^. Wearables for remote digital health and physical activity monitoring have been validated in various settings^11–13^. Given the ubiquity of physical activity monitors, it is surprising their effective incorporation into clinical care remains a challenge, especially in face of the multiple known health benefits of physical activity and the many healthcare scenarios where physical activity information has a clinical use^14–16^. Key challenges preventing inclusion of physical activity monitoring in routine clinical care include the need for data standardization between the many different commercially available devices and sensor locations, and integration of this data into the electronic health record and clinical workflow.

Previous research suggests that digital health programs incorporating health behavior models and personalized coaching are the most successful^17^. Yet, knowledge is limited regarding all the factors that may drive successful implementation of wearables into the healthcare environment^18–20^. Notwithstanding this gap in knowledge, a rapid proliferation of untested digital health applications has led some to dismiss activity monitoring as a health fad that appeals only to fitness fanatics^2,21,22^. Deficits in knowledge have also given rise to concerns that wearables could pose health risks by paradoxically reducing healthy behavior rather than promoting it, either through a false assurance of healthy behavior or through discouragement from failure to achieve goals^2,23–25^. At the same time, many reports indicate a beneficial impact on the health of individuals using wearable monitors^26–30^. Some even indicate that utilization of wearables for clinical care delivery provides health benefits that outpace conventional methods of care^6,31–33^.

These disparate opinions prompted us to seek answers to the current utility of wearables in healthcare. To this end, two of the National Institutes of Health’s Big Data to Knowledge (BD2K) Centers of Excellence—the Mobilize Center at Stanford University^34^ and the Mobile-Sensor-to-Knowledge Center (MD2K)^35^—organized a workshop on the clinical application of wearables. This invitation-only workshop was led by a practicing physician with expertise in the analysis and clinical interpretation of wearable sensor data, and included 14 stakeholders from diverse backgrounds including: hospital administration, academia, insurance, clinical medicine, and the commercial wearable device industry. The workshop group reviewed and discussed two different digital health interventions that have successfully integrated wearables into their clinical workflow, and that empirically demonstrated superior health outcomes using the digital platform compared to traditional care. The workgroup included a representative from each system with direct knowledge of the development and implementation of their respective program. The workgroup’s stated goal was to define the features common to the two sample cases from health systems that allowed the integration of wearables into a successful digital health intervention.

The Ochsner Health System and Kaiser Permanente each designed a digital health program for the management of hypertension and diabetes, respectively^36–38^. In both programs, commercially available at-home digital device (glucometer at Kaiser, blood pressure monitor at Ochsner) was customized to integrate with the electronic health record (EHR) to wirelessly transmit measurements directly to the EHR. Within this framework, wearables were only one aspect of an integrated care delivery process. The digital monitors provide useful instructions directly to patients, while updating the EHR with new data and promoting more rapid and efficient communication between clinicians and patients when needed.

Within the Ochsner system, home blood pressure monitoring and health behavior data from additional optional wearable connected devices (including physical activity monitor and scale), was utilized in conjunction with health coaching and medication adjustments to track patient health status. A mobile application interface served as a feedback loop sending important information to the treating physician, who is then able to seek additional contact if a patient is not meeting expected goals, or simply continue to monitor if all is going well. Within Kaiser, all patients enrolled in the program are actively managed by their care team, who uses the integrated data in EHR to drive clinical treatment decisions, performed in tandem with diet and physical activity education and coaching, and preventive care. This newly tested approach with wearable connected home monitors contrasted from usual care where patients follow-up with care providers in clinic at designated appointments. The lag time in usual care was overcome by the connected digital monitors allowing real-time two-way communication between patient and care team. The new platform also differed from usual care by providing participants with direct technical support to personally address technical issues. It also incorporated a health support team to tackle health-focused problems such as lifestyle management, medication compliance, nutrition, and health education. These teams added a personalized component to each program that specifically addressed important patient-level health needs.

**Fig. 1:**
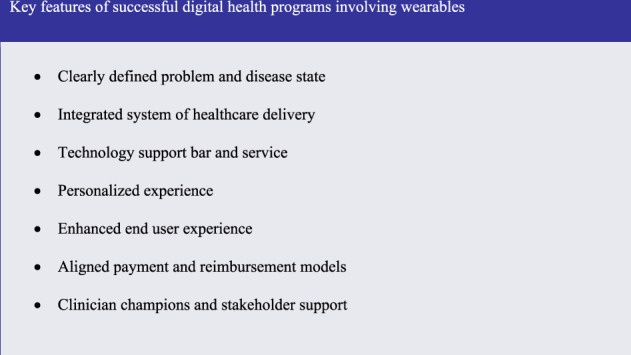
Listed here are the key features identified by the workgroup as responsible for success in digital health programs involving wearables.

Both remote glucose monitoring and blood pressure monitoring digital sensors are commercially available and have been described extensively in the literature^39–42^. The Ochsner and Kaiser Permanent health systems used devices vetted by their own systems and found to be acceptable for use in their patient cohorts. Given the variability in commercially available products and ongoing validation of their output, each health system should carefully review the available options prior to deployment. A 90-day prospective assessment of the Ochsner digital hypertension management program revealed that 71% of patients treated with the digital health program achieved target blood pressure control, compared to 31% under usual care^25^. Kaiser Permanente’s digital glucose monitoring program reduced time for contacting patients in the program via telephone visits by 50%, and therefore effectively doubled clinicians’ capacity to manage patients with diabetes^38^. Both digital programs had similar features that allowed them to empirically outperform usual care. In this paper, we highlight the workgroup’s findings regarding the common key features between these two programs, which underscore the successful implementation of wearables and digital health programs in two different health systems (Fig. 1).

Evaluating the shared characteristics of these programs provides insight into the emerging role of wearable technologies in the clinical arena that expand beyond the role of physical activity monitoring. These shared characteristics provide a roadmap for future implementation of wearables into digital health interventions. Successfully designed and implemented, these new technologies can produce measurable benefits, which accrue to patients and the health system. We suggest that all medical institutions interested in utilizing and scaling wearable technology in their clinical workflow should consider the common features that we outline below. In order to highlight areas with near term potential, we finish by offering our insights into the expanding future direction of wearable physical activity monitoring in the clinical workspace.

## Features for successful implementation of wearables in clinical environments

The workgroup identified many common features between the Kaiser Permanente and Ochsner Health System digital health interventions. Among them, the workgroup agreed that the following common features were directly related to the successful implementation of wearable technology into the digital patient care platform:

### Clearly defined role of wearables to address a specific problem behavior for the disease state

In-line with the scientific method, which begins with a hypothesis, implementation of a successful wearable program requires one to establish a priori goals for the target disease state. In other words, there must be a specific problem that one hopes to address with the wearable technology. As an illustration, Kaiser Permanente and Ochsner identified clinician, patient and system specific problems that were limiting their ability to effectively manage patients with diabetes and hypertension, respectively. For example, clinicians indicated that the frequency and reliability of core disease measures (blood glucose and blood pressure) obtained outside of the clinic were highly variable. Both programs design solutions around this using digital technology applications, including wearables. See Table 1 for an expanded view of the many issues addressed by each program. Simply put, the role of the wearable was clearly defined based on a health-specific need; yet, importantly, the wearable was only one part of a larger digital health program.

**Table 1:** Examples of solutions to problems addressed by each of the seven identified features of successful digital health interventions that include wearables.

Features	Problem(s)	Solution(s)
(1) Clearly defined problem and disease state	*Patient domain*	
	(i) Health literacy	(i) Both programs provided videos and other educational resources on diabetes and hypertension, which were immediately available on mobile phone applications, with automated daily educational emails to patients.
	(ii) Patient adherence	(ii) and (iii) Programs assigned each patient a health coach and also sent daily push notification via phone apps reminding patients to take their medications. Patients were also given access to pill reminder phone apps and where possible, their medication regimen was simplified to facilitate patient adherence.
	(iii) Patient engagement	
	(iv) Social isolation	
	(v) Limited resources/affordability	(iii) Reward points/tokens were provided via mobile apps for completing daily recommended dose of medications and exercises. Apps and wearable monitors provided a feedback loop between clinical teams, health coach, physician and patients for 2-way communication. This allowed immediate feedback on a patient’s clinical status and progress.
	*System domain*	
	(vi) Misclassification bias	
	(vii) Delayed interventions	
		(iv) Patients were able to join digital platforms where they could share daily challenges and progress with hypertension and diabetes management with other patients with similar challenges. Patients are able to develop sense of community with shared goals towards more healthy lifestyle choices.
		(v) Patients who could not afford brand medications were switched to generics or less expensive combination agents, and when appropriate and feasible, enrolled in medication assistance programs.
		(vi) At home monitors helped avoid the issue of white-coat hypertension and allowed more accurate monitoring over the course of 1 week. Patients were encouraged to take 3–4 readings per week. If the care team had not received readings for 8 days, patients received an automated text alerting them that a blood pressure reading was needed.
		(vii) Patients with high digital readings were immediately contacted by health coach or a member of the clinical team to address the problem in real time
(2) Incorporation of wearables into an integrated system of delivery	*Patient domain*	
	(i) Health literacy	(i) and (ii) Home glucose and blood pressure monitoring was linked directly to patients’ EHR. Patient-specific behavioral data were generated in the EHR that allowed a health coach to provide appropriate education on diet and physical activity concordant with a patient’s clinical progress.
	*System domain*	
	(ii) Patient engagement	
		(i) For patients not meeting expected goals, the care team reached out directly to them to address individual barriers and provide focused digital education, as well as automated medication dosing information with human assistance when needed.
		(ii) Health coaches used integrated EHR data to design health and exercise programs, as well as automated text reminders to help patients work toward their goals and to engage them in daily preventive care.
(3) Technology support bar and service	*Patient domain*	
	(i) Limited resources/affordability	(i–iv) Kaiser Permanente and Ochsner each established onsite technology assistance (respectively, the “Thrive Bar” and “O-Bar”) for technology support and troubleshooting. Custom commercial glucose and blood pressure monitoring smart devices were available for purchase at the Thrive Bar and O-Bar. Patients with poor digital literacy received assistance with setting up digital devices and downloading mobile apps on their phones from the tech team. Patient who could not afford smart phones were enrolled in financial assistance programs to help with access to smart devices. Onsite onboarding increased the likelihood of same-day enrollment in the program and shifted the burden of support and technological knowledge from clinicians to the tech support team. This also cut down loss to follow-up.
	(ii) Digital literacy	
	*System domain*	
	(iii) Loss to follow-up	
	(iv) Information asymmetry	
(4) Personalized experience and (5) Enhanced end-user experience	*Patient domain*	
	(i) Digital literacy	(i) The Thrive and O-bars provided a direct a patient-facing service where patients could pick up information, training, and technical support tailored specifically to meet their health needs in addressing challenges with management their diabetes and hypertension.
	*System domain*	
	(ii) Information asymmetry	(ii) Vetted wellness apps by each health system, with gaming aspects were provided as an option to enhance patient adherence. This also provided care teams and patients full information access, further addressing the issue of information asymmetry. It also allowed real-time response from clinicians and care teams. Weekly graphs on glucose and blood pressure values allowed patients and care teams to remotely monitor clinical progress with blood pressure and glucose levels.
	(iii) Loss to follow-up	
	(iv) No shows	
		(i–iv) Patients could call a direct line for assistance with lifestyle management, medication compliance, nutrition, and health education issues. This helped to increase patient engagement and provide information specific to patients’ needs.
		(ii) By linking blood pressure and glucose monitoring devices to patients’ EHR, additional clinical data were obtained from the electronic medical record, including serum sodium, potassium, creatinine, estimated glomerular filtration rate, thyroid function tests, and body mass index (BMI). These data were used to create patient phenotypes, which assisted in the design of patient-specific management plans.
		(iii–iv) To minimize no shows and loss to follow-up, patients in the digital program were given the option for virtual visits vs. face-to-face visits, based on patient preference. An option for an additional wearable activity monitor was also recommended. This provided the patient and physician with important information regarding health behavior adjustments that influence the primary outcome. Allowing the users to opt-in or out of these add-on features is another important aspect of end-user design that fostered digital health program adoption and adherence.
(6) Aligned payment and reimbursement models	*System domain*	
	(i) Fiscal sustainability	(i) Most patients in the Kaiser Permanent system are part of a risk-based or capitated reimbursement models, which provides coverage for chronic health management programs inclusive of digital monitoring devices. Patients in either program that could not afford smart devices were provided medical and financial assistance programs to enable patients to participate in the program. The Ochsner health system self-funded its digital hypertension monitoring program in the initial pilot phase of their program. Subsequently, using collected data from at-risk populations, Ochsner was able to negotiate and establish payment models for their digital health intervention. The CMS Current Procedural Terminology (CPT) code 99091 allows unbundling of billing for chronic care services from billing for the collection and interpretation of physiologic- and patient-generated digital health data. There are specific guidelines for reimbursement, and providers meeting the requirements are reimbursed up to $60 per month per patient for a cumulative 30 min of collecting and interpreting data. Other health systems may tailor digital health program in a way that is fiscally sustainable. For example, health systems may encourage patients to consider opening health savings account, which they could use to fund components of a home digital health monitoring program.
(7) Clinician champions and stakeholder support	*System domain*	
	(i) Program optimization	(i–ii) Kaiser Permanent and Ochsner identified physician champions at local clinical sites to beta test the digital health program, provide feedback, and optimize initial clinical use. After initial buy-in, program optimization and pilot demonstrations of the program’s effectiveness, the health systems scaled the program to other sites. Ochsner’s program primarily targeted primary care physicians (PCP), while Kaiser’s targeted clinical care managers. In the Ochsner program, indirect financial incentives for participation was provided by connecting 5% of physician base compensation to quality metrics. Beyond financial compensation, an internal assessment of physician attitudes about the digital hypertension management program revealed that physician adoption was motivated more by the improved efficiency of care than by any perceived financial reward or incentive. At Kaiser, clinicians are tracked on their diabetes performance by monitoring improvements in HbA1c, “touches” (clinical contacts or treatment intensifications), and according to the Healthcare Effectiveness Data and Information Set (HEDIS) measures/ There was no direct financial compensation tied to performance.
	(ii) Program implementation and stakeholder buy-in	

The left column lists the seven common features identified in the two successful digital health interventions that involve wearables. The middle column outlines problems within the patient domain that are addressed by each feature. The right column lists specific representative examples of solutions provided for these problems by the two sample digital health programs.

### Incorporation of wearables into an integrated system of delivery

Wearables were incorporated into specialized integrated digital care delivery systems, involving health coaching, automated appointment reminders, disease-focused digital education, and automated medication dosing with human assistance when needed^36^.

In both programs, an at-home digital device (glucometer at Kaiser, blood pressure monitor at Ochsner) was customized to integrate with the electronic health record (EHR) to wirelessly transmit measurements directly to the EHR. Within this framework, and as previously described, wearables serve as only one aspect of the integrated care delivery process, allowing the application to provide useful instructions, while updating the EHR and promoting more rapid and efficient communication between clinicians and patients when needed. Within the Ochsner system, home blood pressure monitoring and health behavior data from wearable connected devices, together with health coaching and medication adjustments, combine to provide useful patient health status information to the treating physician, who is then able to seek additional contact if a patient is not meeting expected goals, or simply continue to monitor if all is going well. Within Kaiser, all patients enrolled in the program are actively managed by their care team, who uses the integrated data to drive clinical treatment decisions, performed in tandem with diet and physical activity education and coaching, and preventive care.

### Patient-facing technology support service

Taking a cue from the Apple Store’s “Genius bar,” which is a physical store to assist customers who wish to initiate use of a new Apple device or troubleshoot problems with an existing device, Kaiser Permanente and Ochsner each established onsite technology assistance (respectively the “Thrive Bar” and “O-Bar”) and processes for technology support and troubleshooting. For example, the Ochsner O-Bar is physically located within the medical clinics where staff electronically receive the doctor-recommended digital health intervention and personally assist the patient with selection and purchase of the connected wearable(s), initiation of the health application, and onboarding the application and devices. This increases likelihood of same-day enrollment in the program and shifts the burden of support and technological knowledge from clinicians to the tech support team that is better equipped to help. In addition, the tech team troubleshoots problems with the application and connected devices if they arise, and they provide information and support to patients interested in using additional wellness apps, wearables and connected home devices. All patients who initiate the program are referred to the program by a treating physician, are required to have a smartphone and at least one connected health device (glucometer for the Kaiser program vs. blood pressure monitor for the Ochsner program). The technical support increases patient access to the primary digital health application in addition to other vetted health apps and approved wearables that integrate with the digital health intervention, they facilitate the successful initiation and implementation of the digital health intervention, and ultimately they promote patient engagement and appropriate use of wearables in the clinical setting.

### Incorporation of a personalized human experience alongside the wearable technology

The Kaiser and Ochsner digital health programs each include a human element in the care model and do not rely solely on digital technologies. Of course, all patients continue active management of their overall health with their primary physician. With regard to the focused area of the digital health platform, a human element is also involved. Just as the technical support bar personally addresses technical issues, health support teams are included in each program to tackle health-focused problems such as lifestyle management, medication compliance, nutrition, and health education. A health support team includes: health coaches, nurses, allied health professionals and pharmacists, all with training in the specific chronic health condition for which the program has been designed. These teams add a personalized component to each program that specifically address important patient-level health needs, and in a personalized way that is not provided by current wearable technologies. Development of personalization strategies into digital health technologies amplifies the impact of the digital tools and is an area ripe for innovation in order to reduce the human burden in future applications. By potentially increasing user engagement, personalization may help reduce the observed tendency to abandon use of wearables due to wearables “fatigue” after 3–6 months. Health systems interested in a successful digital health intervention should expect to initially invest in staff training to support patients between clinic visits and to encourage behavioral changes that will foster improved outcomes.

### Focus on the end-user experience

Analysis of factors influencing technology adoption rates indicates that convenience of use and a pleasant end-user experience are critical elements. In the case of wearables in digital health interventions, apps and devices must be designed with both the clinician and patient users in mind. This was the case during the design and testing phases for each of the digital health programs reviewed by the workgroup. Clinicians are interested in easy access to relevant clinical data, efficient integration of the digital platform into the clinical workflow, and ultimately improved treatment outcomes. At Kaiser, clinician feedback was elicited formally through interviews as well as a survey. Both found that the program was well-received by clinicians, where the majority felt that it improved their ability to provide care and that it saved them time^43^. For patients, the application’s usability and design are important for initial adoption, as are the potential for fewer clinical visits and thus fewer co-payments along with reduced time invested in appointments and travel to clinics. Over time, for both clinicians and patients the convenience of use is a motivating factor.

Each of the sample digital programs established minimum requirements and beyond that provided options based on user preference. For example, in Kaiser Permanente’s remote glucose monitoring program, enrolled participants have the option for virtual visits vs. face-to-face visits, based on patient preference. In regard to the use of the wearable device, both Kaiser and Ochsner required a home connected device since it was required for measurement and reporting of the digital health intervention’s primary outcome (blood glucose and blood pressure, respectively). At Ochsner, an additional wearable activity monitor was also recommended, but not required since it was not the primary outcome, yet it provided the patient and physician with important information regarding health behavior adjustments that influence the primary outcome. Allowing the users to opt-in or out of these add-on features is another important aspect of end-user design. In fact, better user experience ratings were revealed in the Ochsner digital hypertension health program when compared to usual care, and this was one factor leading to significantly better improvements in patient engagement and ultimately improved hypertension management^25,32^. This indicates that designing digital health platforms with the user experience in mind is an important aspect of program adoption and success.

### Clearly defined reimbursement model

Establishing a reimbursement model in the design of the digital health program is critical for fiscal sustainability and long-term success. As a combination health insurance and healthcare provider with most patients in either risk-based or capitated reimbursement models, Kaiser’s program is able to be fiscally sustainable as long as the care provided is clinically equivalent or better, provided improved efficiency, and with equal or better patient satisfaction. It was less straightforward for Ochsner, but no less beneficial. Ochsner self-funded its digital hypertension monitoring program in the initial pilot phase of their program, and afterward performed a proof of concept study in at-risk populations. As a result of this initial investment, they were able to provide health payers with data showing significant improvements in patient health outcomes with reduced overall care costs; thus, they were able to negotiate and establish payment models for this digital health intervention. Other systems may take a similar approach or determine alternate strategies toward sustainable reimbursement models. Recently, the Centers for Medicare & Medicaid Services announced the “Physician Fee Schedule Final Rule,” which activates Current Procedural Terminology (CPT) code 99091. This allows unbundling of billing for chronic care services from billing for the collection and interpretation of physiologic- and patient-generated digital health data. There are specific guidelines for reimbursement, and providers meeting the requirements are reimbursed up to $60 per month per patient for a cumulative 30 min of collecting and interpreting data. This new reimbursement model went into effect at the beginning of 2018 and it remains to be seen how this will transform the clinical utilization of wearable device data in healthcare.

### Select data sources that fit into existing data architecture

Since health systems vary by structure and functionality of health data infrastructure, selected data sources must be compatible with existing data architecture. Both Kaiser Permanente and Ochsner had established data streams in their electronic health record (EHR) and worked to design their remote digital health monitoring program to fit existing data structures, and to integrate the program into the existing digital clinical workflow. Oschner used the EHR’s device integration platform to connect to the built-in patient portal, while Kaiser worked with 3rd parties to develop a direct integration that that bypassed the EHR’s patient portal. Patients eligible for the digital health platform by clinical criteria had to next meet technical criteria, which required possession of a smartphone, and then were provided (at Kaiser) or had to be willing to purchase a wireless home monitor (at a discount at Ochsner) from preselected vendors. Stemming from the system design, at Ochsner, patients were required to establish a patient portal account, which they could log into at home to receive and send messages and to access information not delivered through the smartphone application. At Kaiser, the patient portal was optional but encouraged in order to ease communications with the care team. This setup expands the capabilities of the program beyond a simple collection and distilment of data from the connected device and wearable device. It supports two-way communication within a program that includes relevant information about patient comorbidities and other clinically relevant variables already contained in the patients’ charts, and make it available to the patient and the entire care team. Patients and physicians are more effectively connected to the health delivery system via apps, home-based devices and wearables. This provides higher patient engagement in both passive and active formats, and it produces clinically meaningful real-time feedback between patients and their care teams.

### Program deployment with physician champions and opt-in program expansion

Reviewing the Kaiser Permanente and Ochsner programs revealed that clinician champions are important for program adoption and success. Each system identified at least one physician champion at a single clinical site who was keen to beta test the digital health program, provide feedback, and optimize initial clinical use. The champion also served as the most productive clinician in the early rollout of the program and pilot demonstrations of the program’s effectiveness. From here, the program was made available to the other clinicians at the physician champion’s site on an opt-in basis. Once established with buy-in from multiple providers at the original clinical site, the health system began to scale the program to other sites. Ochsner’s program primarily targeted primary care physicians (PCP), while Kaiser’s targeted clinical care managers, and motivations for clinician adoption of the programs were also considered. For example, at Ochsner indirect financial incentives for program use do exist. Specifically, 5% of physician base compensation is tied to quality metrics. For PCPs, hypertension management is among these measures. Since patients using the digital hypertension management system were shown to have better hypertension outcomes than those receiving conventional care, this provided an additional incentive for PCPs to get on board. Interestingly, an internal assessment of physician attitudes about this program reveals that physician adoption was motivated more by the improved efficiency of care than by any perceived financial reward or incentive. At Kaiser, clinicians are tracked on their diabetes performance by monitoring improvements in HbA1c, “touches” (clinical contacts or treatment intensifications), and according to the Healthcare Effectiveness Data and Information Set (HEDIS) measures; however there is no direct financial compensation tied to performance.

## Future opportunities

Health systems like Kaiser Permanente and Ochsner have demonstrated feasibility and successful implementation of digital health monitoring programs that incorporate wearables, with significant improvements in health outcomes compared to usual care. We think the common features identified by our workgroup, and described in this paper, will help guide clinicians and health systems to develop additional programs. As technology continues to evolve, these technologies will become an increasingly integral tool for chronic disease management, health maintenance, and disease prevention. Proceeding while aware of past lessons will help move this technology forward at a faster pace. Recently, many authors have decried the limitations in the implementation and utility of wearable technologies into clinical medicine^44–46^. Here, we chose to highlight the potential and the features common to two current examples of success.

Based on the key features identified above, concerns about the potential paradoxical impact of wearables on healthy behavior appear less significant, especially when wearable information is provided in the context of a comprehensive healthcare augmented by a digital health platform. Wearable sensors and the data they provide are increasing and are expected to become more prevalent in future medical care applications. As demonstrated by the workshop organized by two of the National Institutes of Health’s Big Data to Knowledge (BD2K) Centers of Excellence—the Mobilize Center at Stanford University^34^ and the Mobile-Sensor-to-Knowledge Center (MD2K)^35^—the future remains promising for wearables in healthcare. Integration of wearable sensors into clothing and footwear will eliminate compliance limitations. Addition of new sensors, improvements in the accuracy of the sensor measures, and expansion of clinical insights from wearable sensors will increase their use in the medical environment.

For example, using the standard activity monitors described above, research teams are actively uncovering digital health markers beyond step counts, calorie counts, and minutes of moderate to vigorous physical activity^6^. This has led to new insights into musculoskeletal disease mechanisms^31,47^, and improved rehabilitation through disease-specific physical activity guidelines^48^. This recent work in the musculoskeletal realm has created the instruments and insights necessary for explorations into digital biomarkers of disease using wearable physical activity monitors^49^. We predict this will lead to new tools for disease diagnosis, tracking disease progression and response to treatment, and ultimately predictive models supporting patient-specific precision care.

Implementation of digital health monitoring programs described herein should be tempered by some inherent limitations. First, digital health monitoring programs may be accessible to only a small subpopulation of patients, specifically those who are insured, have access to wireless technology and smart phones, as well as integrated health systems with EHRs linked to mobile phone apps. Some patients in remote and hard-to-reach rural areas may be limited by wireless data capacity, yet these individuals can often benefit most from remote digital health monitoring programs. Therefore, health systems may consider providing financial assistance programs to enable access to smart phones and digital sensors for patients in need. There is also a sampling and selection bias with implementation of digital health programs in that patients who are more-health-conscious are more inclined to participate. In the Ochsner and Kaiser Permanent programs, only patients with a smartphone were eligible to enroll, which further raises questions about education, socioeconomic, and motivational biases. Informative missingness is also a potential limitation from the perspective of enrollees who dropped out early and did not contribute to the success rate of the program. Finally, deployment of these programs requires health system investment into the technology required to transfer, store, analyze and package the digital information and data in a HIPPA compliant manner. Despite these limitations, lessons from these two cases of successful implementation of digital health programs may prove useful to health systems interested in developing or deploying their own programs.

## Conclusion

Utilization of wearables in healthcare environments is expected to increase. As the technology advances, interest will expand. A multi-stakeholder workgroup was convened by two of the National Institutes of Health’s Big Data to Knowledge (BD2K) Centers of Excellence—the Mobilize Center at Stanford University^34^ and the Mobile-Sensor-to-Knowledge Center (MD2K)^35^—to evaluate the clinical application of wearables. Reviewing two effective digital health platforms that incorporate wearables, the group outlined the key factors for successful implementation, including: defining specific problems for targeted wearables solutions in specific disease states, incorporating wearable technology solutions into integrated health systems and existing health data infrastructures, focusing on end-user experience, considering reimbursement models inclusive of digital health programs, and establishing partnerships with clinician champions for wearables in the health ecosystem. Overall, wearables herald a new era in healthcare delivery with the potential to transform many aspects of clinical care.

## Data Availability

This is a qualitative assessment with all of the workgroup’s output contained within the descriptions provided in the manuscript.
